# C-754-07. Pamidronate Dose Response for Hypercalcemia in Burn-Injured Patients

**DOI:** 10.1093/jbcr/irag033.035

**Published:** 2026-04-06

**Authors:** Jason Heard, Gisoo Imani, Erin L Louie, Sierra R Young, Samantha VanAcker

**Affiliations:** Firefighters Burn Institute Regional Burn Center - University of California Davis, Sacramento, CA; Cedars-Sinai Medical Center, Los Angeles, CA; University of California Davis, Sacramento, CA; University of California Davis, Sacramento, CA; University of California Davis, Sacramento, CA

## Abstract

**Introduction:**

Hypercalcemia after burn injury is an underrecognized complication of prolonged hospitalization. Proposed mechanisms include immobility-related bone resorption, cytokine-mediated hypoparathyroidism, glucocorticoid upregulation, and metabolic acidosis. Hypercalcemia may contribute to neuropsychiatric, renal, and cardiovascular complications, yet management in burn patients is not well defined. Pamidronate, a bisphosphonate used for malignancy-associated hypercalcemia, has not been systematically studied in burn-injured patients. This study evaluated its safety and efficacy in this population.

**Methods:**

A retrospective cohort study was conducted of adult burn patients admitted to a regional burn center (October 2013–October 2023) who received intravenous pamidronate for hypercalcemia. Patients with malignancy or end-stage renal disease were excluded. The primary outcome was absolute and percent change in iCa at days 7 and 14 post-treatment. Secondary outcomes included hypocalcemia (iCa ≤1.05 mmol/L), calcium replacement, acute kidney injury (AKI), and renal replacement therapy (RRT).

**Results:**

Twenty-seven patients (37 doses) were included. Mean age was 44 years, 85% male, with mean burn size 60% TBSA. Pamidronate was administered on average 83 days after admission. Baseline iCa was 1.45 mmol/L. At day 7, iCa decreased by 0.26 mmol/L (17.1%), with a similar reduction at day 14 (0.26 mmol/L; 17.0%). Seven patients received additional doses, most >30 days apart. Hypocalcemia occurred after 8 doses (22%). Calcium replacement was given in 35% of cases. No AKI occurred. Patients on RRT had slightly greater iCa reductions, though hypocalcemia post-treatment was more common.

**Conclusions:**

Pamidronate 30 mg IV effectively reduced ionized calcium in burn-injured patients, with effects evident by day 7 and sustained to day 14. Treatment appeared safe across a spectrum of renal function, including RRT, with no new kidney injury observed. Hypocalcemia occurred in about one-fifth of doses, particularly when baseline iCa was <1.35 mmol/L, highlighting the importance of careful patient selection.

**Applicability of Research to Practice:**

These findings suggest pamidronate 30 mg IV is a safe and effective option for hypercalcemia management in burn patients, including those with renal impairment. Clinicians should confirm true hypercalcemia before administration, monitor calcium levels closely, and anticipate supplementation needs. Incorporating pamidronate into standardized protocols may improve management of this complication while minimizing risks, and future research should examine its role in mitigating long-term bone loss and fracture risk post-burn.

**Funding for the study:**

N/A.

